# Higher-order organization of biomolecular condensates

**DOI:** 10.1098/rsob.210137

**Published:** 2021-06-16

**Authors:** Charlotte M. Fare, Alexis Villani, Lauren E. Drake, James Shorter

**Affiliations:** ^1^ Department of Biochemistry and Biophysics, and; ^2^ Biochemistry and Molecular Biophysics Graduate Group, Perelman School of Medicine at the University of Pennsylvania, Philadelphia, PA 19104, USA

**Keywords:** biomolecular condensate, membraneless organelle, liquid–liquid phase separation

## Abstract

A guiding principle of biology is that biochemical reactions must be organized in space and time. One way this spatio-temporal organization is achieved is through liquid–liquid phase separation (LLPS), which generates biomolecular condensates. These condensates are dynamic and reactive, and often contain a complex mixture of proteins and nucleic acids. In this review, we discuss how underlying physical and chemical processes generate internal condensate architectures. We then outline the diverse condensate architectures that are observed in biological systems. Finally, we discuss how specific condensate organization is critical for specific biological functions.

## Graphical abstract

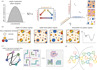

## 1. Introduction

Thousands of biochemical reactions are occurring in a single cell at any given second [1]. Cells therefore need to be efficient multi-taskers to survive, requiring precise spatial and temporal regulation of internal activities. One way that cellular organization can be accomplished is through the use of membrane-bound organelles to group molecules that perform specific functions [2]. Another way that cells achieve internal organization is via liquid–liquid phase separation (LLPS), in which the physical and chemical properties of certain molecules cause them to preferentially self-associate, separating from the cellular milieu [3–12]. These phase-separated condensates, termed membraneless organelles, are formed by proteins and nucleic acids and are linked to a variety of cellular activities, as well as many pathological states [3–15].

As the interest in LLPS in a biological context has increased, so too has the complexity and depth of the questions being asked about the nature and organization of biomolecular condensates. Through efforts to understand the role of LLPS in living systems, researchers have found that many biomolecular condensates are not homogeneous and instead have an internal architecture that has a basis in the nature of their formation and significant functional implications [6]. In this review, we discuss the physical and chemical basis for diverse internal condensate organizations, outline the organizational variation observed in biology and highlight how organization within membraneless organelles can influence function.

## 2. The underlying chemical and physical basis for internal organization of condensates

The phenomenon of LLPS in a cellular context is influenced by a number of fluctuating chemical and physical variables. Due to the highly dynamic nature of the interactions that underlie LLPS, it can be useful to create simplified models to query how interactions between specific molecules change based on their characteristics. These models can take two forms: computational and chemical. In a computational approach, combining the basic principles of polymer physics with experimental data enables the postulation of testable frameworks, which can predict the behaviour of molecules of interest in biological environments [16]. Similarly, targeted manipulations to simplified chemical systems with synthetic or recombinant proteins and nucleic acids can provide deep insight into how particular properties affect in-cell function [17,18]. Together, these basic modelling approaches have been instrumental in describing, predicting and controlling biological processes related to LLPS. First, we will focus on how modelling approaches have revealed the physical and chemical tenets that engender internal condensate organization.

### 2.1. Modelling LLPS for complex systems

**Figure 1.**
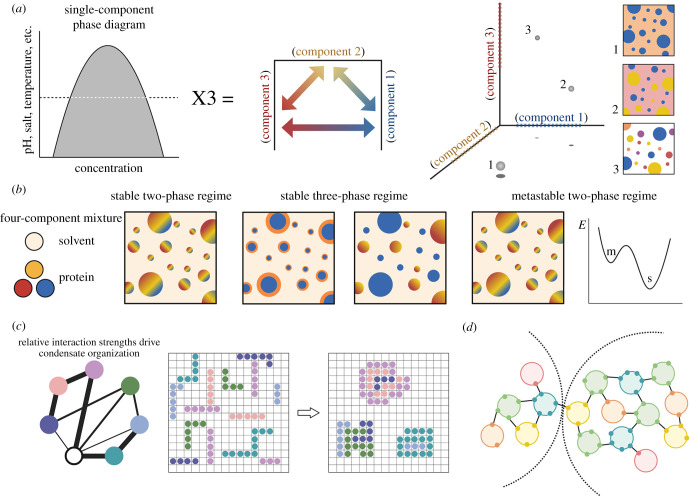
Physical and chemical principles underlie the internal organization adopted by biomolecular condensates. (*a*) In a classic single-component phase diagram (left), the *x*-axis represents protein concentration, and the *y*-axis represents another variable, such as temperature or salt concentration. However, in biological systems, proteins are not typically in isolation. Therefore, the phase behaviour of any given protein is affected by the properties and abundance of the other proteins in its environment. A simplified schema to consider the interplay between three different protein components in a given environment (represented on each axis, where the dashed line on each axis represents the concentration at which a given component will phase separate within a shared environment) is modelled on the right. As illustrated, variations in the concentration of each protein affect which proteins are condensed, whereas other chemical factors influence whether two protein condensates are miscible. In condition 1, component 1 (blue) is condensed, and components 2 (yellow) and 3 (red) are mixed in the light phase (orange). In condition 2, component 3 remains in the light phase, whereas components 1 and 2 form independent condensates. In condition 3, all components are condensed; in addition to each component forming a homogeneous condensate, components 1 and 2 can each form a heterogeneous condensate with component 3 (purple and orange, respectively). (*b*) In addition to coexisting condensed phases, complex mixtures can also form ordered condensates. For example, in a four-component mixture in which two components repel each other, there are three potential regimes. The first is a stable two-phase state in which three of the four components form miscible condensates. The second state is a stable three-phase regime, in which two of the three condensed components are miscible with each other, whereas one is separate. The characteristics of this state depend on the relative concentrations of the components and their interactions with one another. The third regime that can arise from a four-component mixture is a metastable state, which superficially resembles the stable two-phase state. However, a metastable state can only exist in the absence of energetic noise. On a free-energy diagram, this state exists at a local energy minimum (m), but with thermal perturbations, the mixture will shift towards a more stable state (s). (*c*) To understand how condensates form, many researchers have used computational approaches, including lattice modelling [24]. In a lattice model, proteins are represented as polypeptide chains, and the interactions between proteins and with solvent can be parameterized within the simulation. Then, protein chains will self-associate or segregate based on these chemical attributes. Lattice modelling can be useful as both a descriptive and predictive tool for studying biomolecular condensate behaviour. (*d*) One way to conceptualize condensates is as a network of particles in which each particle has some valency [25]. In such a network, components can be classified based on their valency, where a valency of one prevents further network growth, whereas bridges and nodes (capable of forming 2 or 3+ interactions, respectively) promote condensate expansion and association. In this framework, a condensate only forms when there are sufficient cross-links between network particles. The extent to which two condensates interact can be determined by identifying shared nodes between networks. This figure was made with BioRender.

LLPS is a thermodynamic, nonequilibrium process in which a well-mixed state transitions to a demixed state [19]. While the physics of basic binary and ternary mixtures are well characterized, higher-order physical models are required to understand the many components of a cell [20–22]. One approach for characterizing liquid systems with more than one component is a random matrix approximation, a method of estimating molecular interaction strength by generating a matrix of values from a probability distribution [23,24]. However, a relevant physical model for biological phase separation must also account for interactions between phases (figure 1*a*).

To create a formal methodology for describing complex multi-phase interactions, Mao *et al*. [26] modelled a liquid system with four or five components to depict the formation of coexisting phases. They first simplified intracellular fluid by using the Flory-Huggins theorem of regular solutions, which uses interaction strength to determine whether components will mix or repel each other [26]. To define the microstructure of phase-separated condensates, Mao *et al*. focused on the dynamic evolution of the interfacial energies between coexisting phases. For the interfaces formed in a four-component system, in which one component is the solvent, Mao *et al*. established that when a triple-phase junction is mechanically stable, the components form distinct compartments. However, when they are unstable, the phases combine into layered structures. If the interactions between phases are all equal, each component separates to form coexisting phases. On the other hand, if two components strongly repel each other within the four-component mixture, it may form a stable two-phase system, a stable three-phase system or a metastable two-phase regime (figure 1*b*). Thus, in biological systems, variations in interfacial surface tension enable the formation of ordered condensates. As multi-component systems evolve over time, more complex architectures can also form, depending on phase–phase interactions [26].

Lattice models are another framework that can be applied in simulations of multi-valent protein phase transitions. For example, using the sticker-and-spacer domain architecture of protein and RNA molecules, one can parameterize polymer segment interactions (figure 1*c*) [16,27–30]. This approach is adapted from work in associative polymers, where stickers form intermolecular cross-links and spacers can support or prevent the formation of cross-links [29–31]. In molecular biology, protein ‘stickers’ are regions or residues of sequences that facilitate inter- and intra-molecular interactions, whereas ‘spacers’ are often intrinsically disordered and help regulate self-association [29–31].

Choi *et al*. [29] describe one lattice model-based approach, named LASSI (lattice simulation engine for sticker-and-spacer interactions), which generates a coarse-grain model for interactions between multi-valent proteins. LASSI was applied to a multi-component system composed of N130, a truncated form of a nucleolar protein, nucleophosmin 1 (NPM1), and an arginine-rich rpL5 peptide, which undergoes phase separation via heterotypic interactions with N130 [29]. This two-component branched system forms a phase boundary defined by the relative concentrations of N130 and rpL5, yielding a multi-dimensional phase diagram. The generation of multi-dimensional phase diagrams has significant implications for the field of biomolecular condensates, as it reveals that the concentration at which a single particle will phase separate varies as a function of the concentration of other components in the mixture. While the simplest phase separation models assume that saturation concentration (C_sat_, i.e. the concentration at which a molecule will partition into a dense phase) is constant, a multi-dimensional model suggests that the resident molecules in heterogeneous condensates have dynamic C_sat_ values [27,29,32]. Indeed, as LASSI predicts, NPM1 has a variable C_sat_ for nucleolar partitioning [32].

In addition to modelling multi-valent, multi-component protein systems, lattice modelling can also be employed to study the architecture of specific condensates, providing information about how interactions between molecules generate internal organization. Using a simplified system where protein and RNA molecules are represented as strings of interacting modules, Fei *et al*. [33] describe a minimalist lattice model which reproduces the multi-layered organization of nuclear speckles (discussed further below). They found that the strength of specific domain interactions dictated whether nuclear speckles formed a layered structure. Under one binding regime, interfacial tension between the components generated a demixed core and shell. However, altering the binding parameters to reduce interfacial tension generated a mixed, uniform condensate. Thus, lattice modelling is a helpful tool to study multi-component biomolecular condensates. Moreover, because lattice modelling can define variables such as interdomain interactions and percolation thresholds, such models can be used to explain and predict the formation and dispersal of complex phase-separated bodies [34].

While it is understood that the physics of LLPS guides droplet formation, the molecular details of their composition and multi-phase patterning requires further illumination. Borrowing from work in patchy colloids and graph theory, individual proteins and RNAs can be modelled as a system of particles that phase separate depending on their interaction valency [35]. Using stress granules (SGs) and processing bodies (PBs) as a case study, Sanders *et al.* [25] describe how competing protein-RNA interaction networks might control multiphase organization. They found that relative node linkage guides multi-phase organization via a protein–protein interaction (PPI) network. In a PPI network, if a protein can interact with two other partners, it acts as a bridge; if a protein can act with at least three partners, it is considered a node within the condensate network. Thus, a biomolecular condensate network requires the incorporation of bridges and nodes to link the component particles (figure 1*d*). Using such a PPI network, it was demonstrated that tuning the interactions of shared components within the system can regulate multi-phase networks [25]. Similarly, simulating a multi-component system using coarse-grained modelling reveals that condensate stability depends on both the number and orientation of nodes among the component proteins [36]. Moving forward, graph theory represents a powerful tool that can be used to describe multi-component and interacting coacervate systems.

### 2.2. Protein and RNA as organizational scaffolds

Protein and RNA phase transitions organize cellular biochemistry [18,37–42]. Certain protein and RNA molecules function as scaffolds and are necessary for organizing the formation of specific condensates [42,43]. Scaffolds recruit client molecules, which are not sufficient for condensate formation on their own, but associate with condensates due to direct interaction with scaffolds [43]. Together, scaffolds and clients can govern and organize the composition of biomolecular condensates.

To study how protein interactions guide the composition of phase-separated cellular bodies, Banani *et al*. [44] designed a model system using polySUMO (small ubiquitin-like modifier)/polySIM (SUMO-interaction motif) scaffold proteins with monovalent, GFP-labelled SUMO and SIM clients. They found that the polySUMO/polySIM scaffold proteins organized into synthetic condensates, and clients were recruited into them. However, the extent of client association depended on the relative concentration of the scaffold modules. Moreover, yeast P bodies and mammalian promyelocytic leukaemia nuclear bodies (PML-NBs) can regulate client association by using post-translational modifications to vary the number of scaffold interaction sites accessible to client molecules [44].

The oligomerization of several archetypal scaffold proteins has been described in non-synthetic systems as well. In these cases, specific domains between scaffold molecules promote self-association, leading to LLPS. This scaffold-based assembly then enables the accumulation of additional clients. Such a sequential model of scaffold LLPS followed by recruitment of additional factors has been proposed to facilitate transcription [45,46] and other enzymatic processes [47–49]. For example, the protein p62/SQSTM1 condenses in response to various stressors, which promotes the formation of autophagosomes. P62 acts as a scaffold by first oligomerizing via interactions between its PB1 domains, whereas additional interaction domains facilitate the recruitment of other autophagosomal factors [49–51]. The ubiquitin ligase SPOP acts as a scaffold in this manner: interactions between the BTB and BACK oligomerization domains of SPOP form an initial condensate, after which additional clients of SPOP like DAXX can be localized for ubiquitination [48,52]. A similar scaffolding mechanism has also been described for the ubiquitination of gene-body nucleosomes, which we discuss in more detail later [47].

Self-assembly can be drastically altered by simple changes to protein sequence [30,53–55]. This phenomenon is especially interesting in the case of lysine and arginine, two positively charged amino acids that can have strikingly different LLPS properties. Although lysine and arginine both carry a positive charge, arginine can also form π–π interactions that mediate protein : protein association [41,56,57]. To further understand the differences between lysine and arginine in the context of protein condensation, Fisher & Elbaum-Garfinkle [58] characterized the behaviour of polymers with varying lengths of lysine or arginine residues in the presence of negatively charged nucleic acid. They found that, when matched for length and environmental conditions, polyR condenses more readily than polyK on a nucleic acid scaffold, and is more viscous and aggregation-prone [58]. Interestingly, when polyR and polyK were mixed, multi-phase droplets formed. PolyR localized to the interior of these assemblies, consistent with the idea that polyR forms a more dense phase than polyK, with a higher surface tension [58]. Surprisingly, when polyR is added to pre-formed polyK : nucleic acid condensates, polyK is supplanted by polyR. In these experiments, polyR invades the polyK condensate and nucleates its own condensation, releasing polyK into the bulk phase [58]. With these studies, Fisher & Elbaum-Garfinkle demonstrate that protein sequence and abundance can guide multi-layered condensate formation, raising the possibility that altering the concentration of protein components as well as their post-translational modifications could provide a mechanism for the cell to regulate the contents of membraneless organelles.

In recent work, Choi, Bevilacqua & Keating also employ a simplified model of multi-phasic condensates formed by polyK, polyR, and polyD peptides to study how RNA accumulation into such condensates affects RNA structure. In a peptide-only system, polyR and polyD form a mixed phase that is surrounded by polyK [59]. When single-stranded (ss)RNA was added to the peptides, it preferentially associated with the inner phase with polyR and polyD, where it was able to displace polyD : polyR interactions to interact with the positively charged arginine sidechains [59]. Double-stranded (ds)RNA, on the other hand, was present in both the inner and outer phases of the polyK-polyR-polyD droplets [59]. This distinct localization of dsRNA is likely due to a number of factors, including that base pairing reduces the available opportunities for making cation–π and π–π interactions with the peptides, as well as the fact that the excess positive charge in the outer phase may be favourable for the relatively dense-charged dsRNA [59]. In addition to sorting RNAs into specific phases based on their structure, the structure of RNA is also influenced by the thermodynamic environment of the phase into which it is partitioned. In the K-rich outer phase (which is relatively protein-poor), RNA is well-structured, whereas in the protein-dense inner phase, RNA duplexes become destabilized [59]. Surprisingly, RNA is even more destabilized at the interior of the layered three-component condensate than it is in a condensate formed by polyR and polyD alone [59]. The authors propose that RNA partitioning in multi-phase condensates directly contributes to the thermodynamic instability of RNA at the inner phase, suggesting the local environment can act as a helicase, even in the absence of any enzymes [59].

The dense phase of a biomolecular condensate can create a microenvironment with internal chemistry that is distinct from the cytoplasm. In some cases, this microenvironment is capable of melting nucleic acids on its own and could behave like a biomolecular filter [18,59,60]. Nott *et al*. further investigated the concept of a condensate-based molecular filter in a system where nucleic acids partition into membraneless organelles containing the disordered N-terminal domain of the DEAD-box helicase DDX4. They found that unstructured ssRNA molecules partitioned into the condensates most strongly, followed by ssDNA [18]. They also found that the droplets exclude nucleic acid duplexes and solubilize single strands [18]. Furthermore, they demonstrated that the interior phase of these droplets is different from the bulk phase of the cell and can act as a passive helicase, unwinding and remodelling nucleic acid without the need for ATP [18]. These findings suggest that RNA molecules can differentially partition into condensates as a function of RNA structure, which may help explain why some condensates show spatially patterned RNA localization.

RNA also regulates the organization of phase-separated condensates [56]. To investigate how RNA modifies LLPS, Boeynaems *et al*. [56] used soft X-ray tomography to study the effect of RNA on the phase transition behaviour of specific dipeptide-repeat proteins (DPRs) connected to amyotrophic lateral sclerosis (ALS) and frontotemporal dementia (FTD). In disease, these DPRs are generated by repeat-associated non-AUG (RAN) translation of an expanded G_4_C_2_ hexanucleotide repeat in the first intron of the *C9ORF72* gene, the most common genetic cause of ALS/FTD [56]. Mixing the C9ORF72 DPR (PR)_30_ with either poly-rA, -rU or -rC RNAs yielded liquid condensates. However, when (PR)_30_ was mixed with poly-rG, which is prone to forming G quadruplexes, (PR)_30_ and poly-rG formed large, fractal-like networks, which are structurally more akin to a kinetically arrested gel-like state. Using protein–RNA-solvent coarse-grain simulations, Boeynaems *et al*. [56] demonstrated that RNA forms stable scaffolds and recruits protein, but RNA–protein interactions compete with RNA base pairing, which can lead to arrested phase separation.

Boeynaems *et al*. [56] also found that RNA affected droplet viscosity, as poly-rA formed more viscous droplets than -rU or -rC. Furthermore, mixing poly-rA and poly-rC RNA in a 1 : 1 ratio caused the formation of multi-layered condensates with a poly-rA dense core and poly-rC labile shell. By increasing the relative abundance of poly-rA, they could form a droplet with inverse RNA organization, where poly-rC formed the core. (PR)_30_ was found throughout the multi-layered condensates, but the DPR was less dynamic in the poly-rA layer than poly-rC layer, regardless of condensate organization [56]. These findings demonstrate differential compartment dynamics, which may be due to the formation of cation–π or π–π interactions between the rA's double aromatic ring and the arginine residues in (PR)_30_.

Clearly, RNA can modulate phase behaviour and the formation of dynamic substructures within droplets. Banerjee *et al*. [61] investigated whether RNA can mediate reentrant phase transitions and drive substructure formation. A phase transition is considered reentrant when alteration of a single thermodynamic variable elicits two (or more) phase transitions that enable the acquisition of a state which is macroscopically similar to the original state in terms of the number of different phases and their respective material properties [62]. Using two synthetic peptides containing multi-valent arginine-rich, low-complexity motifs, it was demonstrated that as RNA concentration increases, the ribonucleoprotein (RNP) system undergoes demixing and subsequent remixing typical of a three-regime phase diagram in which reentrant phase transitions occur [61,63]. Reentrant phase behaviour could also be observed inside these condensates: when additional RNA was introduced into the RNA–protein droplets, coexisting droplets of different densities were formed, demonstrating the ability of RNA influx to drive the formation of dynamic substructures. To further characterize this system beyond simple synthetic peptides, protein–RNA interactions were also studied using FUS, a RNA-binding protein (RBP) with a prion-like domain (PrLD) that is associated with neurodegenerative disease [61,63–65]. Adding RNA to FUS promoted analogous mixing and demixing behaviour to that of the synthetic protein : RNA system. Similarly, adding RNA to FUS : RNA droplets produced a ‘vacuolar’ organization, demonstrating the controlled formation of RNA-driven multi-compartment liquid droplets in a relevant RNP model [61,63].

Considering the role that arginine residues play in LLPS, Kaur *et al*. [66] extended these studies to examine the behaviour of a three-component system consisting of a PrLD, an arginine-rich polypeptide (RRP), and RNA. The behaviour of each of these individual components is affected by the relative concentrations of the other two. Specifically, the presence of an RRP induces PrLD LLPS, whereas RNA can promote LLPS of RRPs but not of PrLDs, underscoring the importance of arginine in forming protein : RNA condensates [66]. These findings may be of relevance in C9-ALS/FTD, where arginine-rich DPRs are particularly toxic [67–74]. Intriguingly, RRP : RNA condensates adopt an internal structure when one component is present in excess over the other, with the more abundant molecule residing on the surface of the condensate [66].

These results suggest that proteins with arginine-rich domains may act as nodes within condensate networks, coordinating interactions between various condensate components. In fact, when PrLD : RRP droplets were made at low PrLD concentrations, the addition of RNA sequestered the RRP away from these droplets, causing the PrLDs to dissociate [66]. However, when PrLD : RRP condensates were formed with high relative PrLD levels, the addition of RNA enabled the formation of RRP : RNA droplets, but PrLD condensates persisted, leading to a coexisting droplet network with distinct RRP : RNA condensates on the surface of the PrLD droplets [66]. In addition to this associative droplet network, Kaur *et al*. [66] could also generate condensate architectures with immiscible layers by varying the ratio of RNA to RRP.

The studies highlighted in this section demonstrate that the chemical and physical environment at interfacial surfaces provides a critical foundation for the construction of more complex condensate topologies. Thus, understanding how such interfaces are modified to affect condensate biology is an intriguing avenue for future research. It is also clear that the relative concentration of various molecules can drive or inhibit condensate structure, in part by limiting access to shared binding partners. Elucidating the mechanisms by which the cell tunes the levels of protein and RNA to generate condensates that are responsive to environmental and developmental changes is another exciting area for research.

## 3. Examples of biomolecular condensates with internal architecture

Research using simplified *in vitro* and synthetic systems has been foundational in characterizing the types of interactions that occur between molecules in condensates. Furthermore, these studies have demonstrated how multi-component condensates found in living systems might adopt an internal organization. In this section, we will highlight general categories of condensate organization and provide specific examples of where these architectures are observed.

### 3.1. Solid core-liquid shell

**Figure 2.**
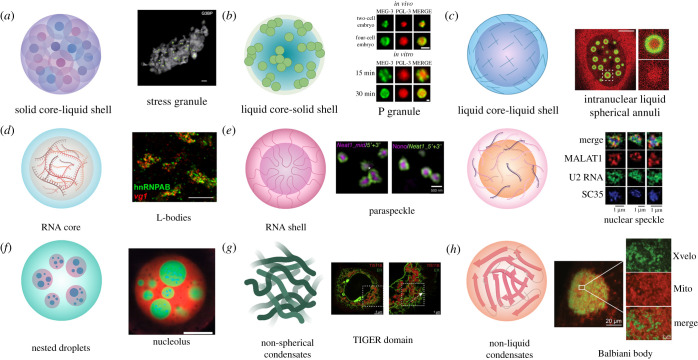
Examples of biomolecular condensate architecture. (*a*) Solid core-liquid shell. Condensates with this structure initially form as individual protein cores, which then accumulate more material and join together to form larger structures, as with SGs. Green dots indicate G3BP cores and grey represents the surface area of the SG; scale bar represents 500 nm [83]. (*b*) Liquid core-solid shell. Condensates with this structure consist of a more liquid-like core surrounded by a gel-like protein shell. An example of this architecture is the P granule found in *C. elegans* embryos. Condensates formed by MEG-3 (green) and PGL-3 (red) in cells and *in vitro* show this architecture; scale bars represent 1 µm [84]. (*c*) Liquid core-liquid shell. With this architecture, the interior and the exterior of the condensate are both dynamic liquids, but are compositionally distinct, such as with iLSA. iLSA have a liquid TDP-43 shell (green) and the interior is enriched for HSP70 chaperones, including Hsc70 (red); scale bar represents 5 µm [85]. (*d*) RNA core. In *X. laevis* oocytes, RNAs must properly localize for successful embryonic development. This localization is achieved, in part, by the formation of L-bodies, composed of RNAs and RBPs. In L-bodies, the RNA forms an immobile core enveloped by a dynamic protein coating. Staining for the L-body protein hnRNPAB (green) and mRNA vg1 (red) reveals the multiphasic nature of these condensates; scale bar represents 10 µm [86]. (*e*) RNA shell. There are multiple ways in which a condensate can form such that protein is surrounded by RNA. In paraspeckles (left), the lncRNA NEAT1 folds so that its 3′ and 5′ ends face outward with its middle at the centre where proteins accumulate. Labelling the 5′ and 3′ ends of NEAT1 (green) highlights the paraspeckle shell, whereas labelling the middle portion of NEAT1 or a protein such as Nono (purple) identifies the core; scale bar represents 500 nm [87]. Alternatively, RNAs can decorate the exterior of a protein core, as with nuclear speckles (right). In this situation, a proteinaceous core collects RNA molecules that reside on the outside of the condensate. SIM images of nuclear speckles show splicing protein SC35 (blue) occupying a central area, with lncRNA MALAT1 (red) and U2 RNA (green) taking up an area with a larger radius; scale bars represent 1 µm [33]. (*f*) Nested Droplets. The nucleolus is an example of a condensate with a nested droplet architecture. The nucleolus has a tripartite organization, with the gel-like FC and DFC enveloped by a liquid-like GC. Each component of the nucleolus has a distinct function and displays distinct physical properties. In *X. laevis* nuclei, RNA Pol I (blue) localizes to the innermost FC, FIB1 (green) stains the DFC, and NPM1 (red) is localized at the liquid-like GC; scale bar represents 20 µm [88]. (*g*) Non-spherical condensates. Biomolecular condensates can also adopt atypical structures, as in the case of TIS11B granules. TIS11B granules form an extensive mesh-like assembly composed of protein and RNA. In cells, TIS11B condensates (red) intertwine with the ER (green), forming a complex network of tubules; scale bars indicate 5 µm (left) and 1 µm (right) [89]. (*h*) Non-liquid condensates. In addition to liquid-like condensates, biomolecules can also condense to form solid states. One example of this is the Balbiani body, which contains an amyloidogenic network formed by the protein Xvelo in *X. laevis* (homologous to the Bucky ball protein in zebrafish). Xvelo (green) contains a PrLD which is necessary for its self-assembly, as well as its association with organelles, like mitochondria (red); scale bars indicate 20 µm and 2 µm (insets) [90]. This figure was made with BioRender.

SGs are cytoplasmic membraneless organelles with a layered internal structure and are found in cells upon exposure to environmental stressors, including heat stress, oxidative stress and chemical stress [10,11,75,76]. In response to adverse conditions, the majority of translation is paused, and RBPs with their associated mRNA transcripts are exported from the nucleus to the cytoplasm, where they condense to form SGs [10,76–80]. A key feature of SGs is that they are dynamic, readily exchanging material with the cytosol [81,82]. Their formation, though, follows a relatively patterned process that establishes a specific internal organization with a dense core and a more dynamic shell (figure 2*a*) [83].

Jain *et al*. [83] established a core-shell model for SGs by comparing stressed and unstressed cell lysates. When subjected to stress, cell lysates contain punctate structures that are positive for many SG markers [83]. However, these structures were smaller than SGs observed in cells, suggesting that they represented SG cores [83]. Super-resolution microscopy of SGs in cells revealed that component proteins were not uniformly distributed throughout the SG ultrastructure, but indeed were concentrated at discrete cores [83]. Proteomic analysis of the proteins at SG cores revealed that these proteins share several properties. First, RBPs and translation factors are abundant at SG cores [83]. Second, many SG core proteins have PrLDs, allowing these proteins form an extensive and cooperative interaction network [83]. Third, there are a high number of proteins with ATPase activity found at SG cores [83] Moreover, ATP is required for maintaining SG fluidity, suggesting that the ATPases found in the SG cores are actively modulating SG physical properties [81]. Thus, SG cores are formed by proteins with an inherent ability to participate in multi-valent networks that can also actively promote ATP-driven remodelling processes.

Investigations into how SGs form revealed that the core-shell architecture of SGs is established temporally [81]. In the lifecycle of SG formation, the SG core appears first: following an initial stressor, SG core proteins are exported to the cytoplasm where they oligomerize and condense [81]. SGs can then grow by incorporating additional materials and joining with other nascent SGs [81]. The formation of the SG core therefore serves as a nucleating event that allows for the formation of the mature SG, which is held together by the interactions between the less dense, more dynamic shells surrounding adjacent cores [81]. Disassembly of SGs proceeds in roughly the reverse order as assembly, where the SG is first separated into smaller foci, which then disappear over time [81]. However, while these cores may seem to disappear, it may be the case that remnants of the SG core that are below the diffraction limit of standard microscopy techniques cannot be resolved, and these may serve as nucleators for future SGs, other condensates or protein aggregates [79,82,91–95].

Studies on SG formation suggest that several different core proteins may serve as SG nucleators. Ras GTPase-activating protein-binding protein 1 (G3BP1), T-cell intracellular antigen-1 (TIA-1), TIA-1-related (TIAR), fragile X mental retardation protein (FMRP) and recently ubiquitin associated protein 2-like (UBAP2L) have all been identified as SG scaffolds that lay the foundation for mature SG formation [25,79,92]. There is likely no single ‘master scaffold’, but instead many proteins with PrLDs and RNA-binding domains (RBDs) can serve in the first step of constructing a SG [76,96–99].

Given the requisite properties of SG proteins, SGs may present a risky strategy for the cell confronting environmental stress. In the short-term, SGs store a wide range of proteins, including those that influence signalling pathways and mRNA metabolism [9,99–101]. In the long-term, though, if mutations to SG resident or modifying proteins prevent SG disassembly, or if chronic stress leads to prolonged and repeated SG formation, the high local concentration of aggregation-prone proteins can lead to the formation of toxic cytoplasmic aggregates [55,75,76,94,95,99,101,102]. Moreover, persistent SGs may trap proteins, preventing them from performing their function and leading to cell death [101]. Investigations into how SG organization changes with time or in different disease states may lead to new insights into the function and properties of SG condensates [103].

PBs are another RNA–protein condensate that forms a dense core with a dynamic shell [104–109]. PBs are constitutively present in the cytoplasm and play a role in translational repression [77,105,109]. Many of the PB-resident proteins are associated with mRNA repression and degradation, including the DEAD-box helicase DDX6 (Rck/p54), RNA exonuclease Xrn1 and the RNA-decapping enzymes DCP1A, DCP1B and DCP2 [104,109]. While some proteins, including Xrn1 and DCP1A, are found most densely at the centre of PBs, others can show a preference for the periphery of the condensate [104,110].

Such differences in protein localization led researchers to wonder if the RNA component of PBs is similarly organized. To answer this question, Pitchiaya *et al*. determined the localization of microRNAs (miRNAs), mRNAs, and long non-coding (lnc)RNAs in PBs by building an intragranular localization atlas of known PB proteins [110]. They found that let-7, an endogenous PB miRNA, docks at the PB exterior, oligomerizes, and becomes less mobile relative to cytoplasmic miRNAs. mRNA targets of let-7, such as FL-l7–6x-MS2, interacted more stably with PBs than mRNAs without let-7-binding sites [110]. Furthermore, upon removing the sequences that are targeted by let-7, FL-l7–6x-MS2 mRNA localized to PBs less frequently, and its translation was significantly increased [110]. Finally, Pitchiaya *et al*. looked at the lncRNA THOR. THOR molecules were found more frequently at the PB shell and interacted more transiently with PBs than miRNAs or mRNAs [110]. Thus, proteins and RNAs at the core of PBs probably represent a stable site of translational repression and degradation, whereas the shell is a site of stochastic association with translatable mRNAs where RNA helicases may play a regulatory role [77,109–111].

PBs and SGs are physically and functionally linked, which results in higher-order condensate systems. In the cell, adjacent PBs and SGs can dynamically interact, allowing for the exchange of RNA and protein [78,112]. Yet, PBs and SGs remain compositionally and spatially distinct, with overlapping but independent proteomes [25,105]. The underlying basis for how PBs and SGs generate bridged condensate networks can be explained by graph theory, which suggests that interacting particles form a network when the component particles are multi-valent (figure 1*d*) [25].

To identify bridges and nodes within cells, it was shown that the deletion of core SG proteins, G3BP1/2, prevents SG formation under specific conditions [25]. Surprisingly, substituting the dimerization domain of G3BP (NTF2) with that of another protein does not recover SG formation, indicating that the native G3BP dimer acts as a node, not a bridge [25]. One way for G3BP to act as a node is if the dimerized NTF2 domains introduce an additional point of valency [25,113,114]. To test this hypothesis, the authors turned to an optogenetic oligomerization tool named Corelets, which can be used to induce the formation of artificial condensates. Corelets are a two-module system that relies on the light-based dimerization between an iLID domain on a ferritin heavy chain protein core and an SspB domain on the molecule of interest [115]. Using Corelet-mediated NTF2 condensates, the researchers identified candidate SG:PB-interaction partners for the isolated domain [25]. Of these candidates, only UBAP2L knockout affected SG formation, suggesting that G3BP dimers form a node with UBAP2L [25]. Interestingly, Corelets with only the RBDs of G3BP can also form stress-dependent condensates in cells, and these synthetic opto-SGs associate with PBs, indicating that under these artificially defined conditions, RBDs can play a necessary role in SG : PB interactions [25]. When tested, Corelets bearing the RBD of other SG proteins also form opto-SGs decorated with PBs [25].

Particles in a condensate system must compete for node and bridge occupancy, and therefore the stoichiometry of molecular constituents influences network formation. If UBAP2L acts as a node between SGs and PBs, then its overexpression would lead to excessive network formation and condensate miscibility [25]. Indeed, UBAP2L overexpression in G3BP knockout cells led to the formation of merged condensates containing proteins found in both SGs and PBs [25]. Furthermore, the overexpression of SG- or PB-specific components results in SG : PB decoupling, due to a relative lack of shared nodes in the network [25]. These findings indicate that the concentrations and relative valency of network particles may act as environmentally sensitive phase switches, where expression levels can modulate phase behaviour [25]. Additionally, by controlling the availability and occupancy of shared nodes, cells might coordinate tasks of independent condensates such as RNA processing.

### 3.2. Liquid core-solid shell

While SGs and PBs are composed of a solid-like core surrounded by a more liquid-like exterior, other membraneless organelles show a reciprocal organization where a liquid centre is surrounded by a solid exterior shell (figure 2*b*). Putnam *et al*. [84] identified an example of the liquid core-solid shell architecture in their study on P granules in *C. elegans* embryos. P granules are liquid RNP condensates found in the posterior half of *C. elegans* embryos, and their biogenesis and proper localization may help to establish the body plan of the developing worm [84,116,117]. P granule formation requires MEG-3, an intrinsically disordered protein that undergoes LLPS [116]. Analysis by fluorescence recovery after photobleaching (FRAP) accompanied by experimental measures of surface tension demonstrated that globally, P granules behave as a liquid in cells [117]. However, further investigation into the nature of P granules revealed that their protein components occupy spatially distinct regions of the granule, leading researchers to propose that P granules are layered condensates [84]. Indeed, high-resolution microscopy showed that MEG-3 resides on the outside of P granules, whereas key P-granule proteins PGL-1 and PGL-3 are found at the interior [84].

To study the dynamics of P-granule components, Putnam and colleagues sequentially fused resident P-granule proteins with GFP and performed FRAP experiments. In live embryos, they saw that PGL-1 and PGL-3 were very dynamic, but MEG-3 was significantly less mobile [84]. Furthermore, when MEG-3 and its paralog MEG-4 were deleted in embryos, PGL proteins were even more liquid-like [84]. This finding suggested that P granules are composed of a liquid-like interior with a more gel-like outer shell. To definitively test this hypothesis, Putnam *et al*. subjected *C. elegans* embryos to rapid temperature shifts to determine the effect of temperature on PGL-3 and MEG-3 self-assembly. They saw that an increase in temperature caused PGL-3 condensates to dissolve, which is expected for a liquid phase; MEG-3 condensates, though, were stable at 34°C [84]. Similarly, when the embryos were punctured, allowing the cytoplasmic contents to be diluted into buffer, PGL-3 droplets dissolved but MEG-3 droplets persisted [84]. Remarkably, MEG-3 droplets were also resistant to buffers containing high salt, ATP or 1,6-hexanediol, which can disrupt liquid compartments and inhibit their formation [118,119]. By contrast, MEG-3 droplets were sensitive to SDS, leading the authors to conclude that MEG-3 forms a stable gel at the P granule surface [84]. MEG-3 forms a shell around PGL-3, which stabilizes PGL-3 condensates, contributing to spatially restricted P granule formation. Because MEG-3 is enriched at the posterior of the *C. elegans* embryo, its localized condensation drives stable PGL-3 : MEG-3 droplet formation. Thus, Putnam *et al*. demonstrate that P granules constitute a liquid-like core surrounded by a gel-like shell in a complex biological system and in simplified biochemical assays. Whether this condensate architecture is also observed in other organisms has not yet been addressed.

### 3.3. Liquid core-liquid shell

Layered condensates can also form as partially immiscible liquids, in which each layer has dynamic properties. Such a layered liquid architecture was described by Yu *et al*. where acetylated TDP-43 forms a liquid shell surrounding a liquid core that is enriched in Hsp70 chaperones (figure 2*c*) [85]. TDP-43 is an RBP with a PrLD and is found in cytoplasmic and nuclear condensates, and in pathological inclusions [65,120]. In neurodegenerative diseases such as ALS and FTD, cytoplasmic aggregates of TDP-43 are highly acetylated in patient tissue, and in experimental models, acetylated TDP-43 is more aggregation-prone [120]. Furthermore, acetylation of TDP-43 occurs in a stress-dependent manner, and precludes TDP-43 from binding to RNA, suggesting that acetylation of TDP-43 tunes its physical and functional state [120].

Because acetylation both reduces RNA binding and promotes TDP-43 phase separation in the cytoplasm, Yu *et al*. hypothesized that intranuclear TDP-43 acetylation would also alter its condensation [85,121]. This hypothesis was based in part on earlier work that demonstrated that reduced RNA binding has profound effects on TDP-43 phase separation [119,121]. For example, Schmidt & Rohatgi replaced the RNA-recognition motifs (RRMs) of TDP-43 with GFP and observed that TDP-43 formed nuclear condensates filled with vacuoles of nucleoplasm. Both the TDP-43 and nucleoplasmic components of these condensates were viscous liquids that exchanged material with the bulk nucleoplasm [121]. Similarly, when Yu *et al*. manipulated the RNA binding of TDP-43 by treating cells with deacetylase inhibitors, or by mutating two lysine acetylation targets to glutamine to mimic acetylation, they saw that nuclear TDP-43 formed spherical shells [85]. FRAP experiments revealed that the TDP-43 molecules within these structures rapidly exchanged with the nucleoplasm. Moreover, using differential interference contrast (DIC) imaging, Yu *et al*. observed rapid fusion of the droplets, suggesting that both the outer shell and its contents behave like liquids. Thus, Yu *et al*. [85] term these unique condensates intranuclear liquid spherical annuli (iLSA) or anisosomes.

To further probe iLSA organization, Yu *et al*. used cryo-electron tomography (CryoET) to study the structure of iLSA within cells. They saw that the shell appeared to be an ordered liquid [85], and posited that TDP-43 may be forming a liquid crystal in which molecules are organized and fluid [122,123]. Liquid crystals are anisotropic, meaning that the component molecules exhibit different refractive indices along a given molecular axis [122]. To determine if, in fact, iLSA are liquid crystals, Yu *et al*. used complete extinction microscopy (CEM) with polarized light, allowing them to use sample brightness as a measure of anisotropy. CEM confirmed that iLSA shells are anisotropic, and that acetylated TDP-43 forms a condensed ordered liquid in the nucleus.

Yu and colleagues also wondered what molecules were found inside iLSA. They discovered that chromatin, as well as some RBPs like FUS, were excluded from the annuli, whereas other RBPs, including hnRNPA2B1 and hnRNPK, were found throughout the iLSA [85]. Interestingly, although iLSA are enriched for RBPs, they are depleted of RNA molecules relative to the nucleoplasm [85]. To develop a theoretical framework for the composition and formation of iLSA, Yu *et al*. used mathematical modelling to simulate RNA-binding-deficient TDP-43 monomers and dimers in the nucleoplasm. In their model, acetylated TDP-43 on its own could de-mix, but was not sufficient to reproduce iLSA formation. Thus, they reasoned that there must be some additional variable present in the nucleus that enables iLSA to form. Further, they hypothesized that such factor or factors would be able to self-assemble and co-condense with TDP-43, but not with RNA, as iLSA only form when acetylated TDP-43 is present. Upon assigning this unknown variable an intermediate affinity for TDP-43, Yu *et al*. were able to successfully model the annuli they observed.

Finally, Yu *et al*. set out to identify the additional factor(s) necessary for their model to recapitulate iLSA formation. To this end, they performed proximity labelling followed by mass spectrometry and found that the five most enriched proteins in the iLSA-positive cells belonged to the Hsp70 chaperone family [85]. Subsequent experiments using fluorescently tagged Hsp70s confirmed that these chaperones are enriched at the iLSA core [85]. Moreover, inhibiting Hsp70 ATPase activity prevented iLSA formation and led to the formation of uniform TDP-43 droplets, indicating that Hsp70 is actively maintaining separate liquid layers [85]. The researchers suggest that perhaps TDP-43 acetylation and subsequent loss of RNA binding exposes an unstable portion of TDP-43 which in turn recruits Hsp70 to assist in iLSA formation. It is unclear, though, why hydrolysis-competent Hsp70 does not fully dissolve the TDP-43 shell, which might be expected as Hsp70 forms part of a human protein-disaggregase machinery [12,124–128].

While RNA is not generally enriched in iLSA, the abundance of RBPs in the structure raises the possibility that specific RNAs might be found in iLSA. However, this study did not look into whether any RNA molecules or RNAs with particular structural motifs display a preference for iLSA inclusion. Thus, rigorous characterization of the protein and RNA contents of iLSA will provide insight into how its structure is maintained and may uncover a physiological function. Intriguingly, some ALS-linked mutations in TDP-43 are found adjacent to the RRMs and reduce RNA binding [129,130], and thus whether iLSA and the RNA-binding capacity of TDP-43 are implicated in disease and ageing warrants further investigation.

### 3.4. RNA core

RNA is often a major component of biological condensates, due to its negative charge and flexible structure, as well as the fact that many RBPs undergo LLPS [131–133]. Understanding the nature of RNA–protein interactions within biological condensates is a burgeoning field, and recent work indicates that subcellular RNA localization facilitated by LLPS is critical for organismal development [84,86,134]. For example, in *X. laevis* oocytes, specific mRNAs must be transported to the vegetal pole for successful embryonic development [86,135]. Localization of these mRNAs occurs through interactions with RBPs and can result in the formation of RNA–protein condensates [86,136]. By performing fluorescence *in situ* hybridization (FISH) with labelled probes that hybridize to vegetal mRNAs such as *vegT*, *trim36* and *vg1*, Neil *et al*. observed that these mRNAs are found in distinct foci in the vegetal cytoplasm, segregated from the RNA molecules found at the outer vegetal cortex [86].

To further characterize these localization bodies (L-bodies), Neil *et al*. isolated the structures from oocyte lysates using size exclusion chromatography and then performed co-immunoprecipitation (co-IP) analysis with known vegetal RBPs including Vera, Stau1 and hnRNPAB [86]. They discovered that each of these proteins is also found in L-bodies [86]. Next, Neil *et al*. performed mass spectrometry on the isolated L-bodies to catalogue their protein composition. Gene ontology (GO) analysis of the L-body proteome revealed that L-bodies are highly enriched for proteins involved in nucleic acid binding and ATP binding [86]. The authors speculate that the presence of DDX helicases may be important for regulating L-body RNA–protein interactions [86]. Of the proteins identified from their mass spectrometry analysis, approximately 25% are unique to L-bodies, providing additional evidence that L-bodies are a novel biomolecular condensate composed of RNA and protein [86].

In examining the L-body proteome, Neil *et al*. noticed that proteins with PrLDs [137] were overrepresented relative to the *X. laevis* proteome, and so they sought to determine if this bias generated distinctive biophysical properties. To address this question, the researchers stained oocytes with thioflavin-T (ThT) to selectively identify the cross-beta strands of amyloids [86]. Upon doing so, Neil *et al*. uncovered a mesh-like substructure to which L-body mRNAs colocalize. Further analysis by FRAP of L-body proteins and RNAs revealed that, while the proteins display highly mobile character, it was the RNAs that were immobile, suggesting that L-bodies contain a solid-like RNA core, rather than a protein amyloid core with a dynamic protein coating (figure 2*d*) [86]. Furthermore, RNA from *X. laevis* oocytes binds to ThT and can form droplets and gels *in vitro*, suggesting that the ThT staining in oocytes is related to a solid- or gel-like RNA structure [86]. In this way, the RNA component of L-bodies acts as a scaffold to recruit proteins that bind to and process the RNAs. Future work to ascertain how L-body formation and dissolution is regulated will be key to understanding the role of these condensates in development.

Condensates with RNA at their core have also been identified in mitochondria, in the form of mitochondrial RNA granules (MRGs) [138]. Mitochondria are dynamic organelles that harbour their own, distinct genome. Thus, when mitochondria undergo dramatic changes in shape, their genetic organization must simultaneously adapt. Newly synthesized mitochondrial RNA (mtRNA) had been previously observed to interact with mitochondrial RBPs to form MRGs, and these structures are important for mtRNA processing [138,139]. To understand how MRGs respond to changes in mitochondrial morphology, Rey *et al*. examined their structure and localization, and found that MRGs display a layered architecture with mtRNA at their core, surrounded by RBPs. Within MRGs, both the protein and nucleic acid components are liquid-like, in stark contrast with the similarly punctate but less mobile nucleoids formed by mtDNA [138], which may be more viscous liquids [140]. Typically, MRGs are randomly distributed across the inner-mitochondrial membrane [138]. Upon blocking mitochondrial fission or fusion, however, the researchers observed that MRGs cluster, but do not fuse, in the resulting mitochondrial compartments [138].

### 3.5. RNA shell

RNA in general can influence phase transitions [119,141], and specific RNAs have well-defined roles in shaping condensate architecture [142]. One such case is that of paraspeckles. Paraspeckles are nuclear membraneless organelles involved in many processes. Paraspeckles act as molecular sponges to regulate the level of active proteins in the nucleus and they are important for gene expression [87]. Paraspeckles form upon transcription of NEAT1, a lncRNA that is transcribed as two isoforms: a short isoform, NEAT1_1, and a long isoform, NEAT1_2 [143]. Each isoform is transcribed as a single transcript, after which NEAT1_1 is polyadenylated, whereas NEAT1_2 folds and is cleaved, resulting in a mature transcript with a non-polyadenylated 3′ end [143]. Both isoforms are retained in the nucleus and are the foundation for paraspeckle biogenesis, with NEAT1_2 serving as the primary architectural molecule [87,143].

Examination of paraspeckle architecture by structured illumination microscopy (SIM) revealed that NEAT1_2 folds such that its middle region is located at the centre of the speckle, with its 5′ and 3′ ends facing out (figure 2*e*, left) [87]. This ultrastructure houses dozens of proteins, which themselves have distinct spatial arrangements [87]. There are core proteins found at the centre of the paraspeckle, which include the Drosophila behaviour and human splicing (DBHS) family of proteins (i.e. Sfpq, Nono and Pspc1) and FUS [87]. Additional proteins are found throughout the paraspeckle, but TDP-43 was the only protein tested that was found to reside at the outer edge of the paraspeckle [87]. In addition to the 5′ and 3′ ends of NEAT1, other RNA molecules are also found at the surface of paraspeckles, including both spliced mRNAs and specific introns [87].

Nuclear speckles, like paraspeckles, are a nuclear condensate composed of RNA and protein [33,144,145]. Nuclear speckles participate in mRNA transcription, splicing, and processing, and are identified by the presence of phosphorylated serine-arginine (SR)-family splicing regulators like SC35 (SRSF2) and the splicing co-activator SRRM2 [33,145]. More detailed research into the structure of demixed nuclear speckles uncovered that mRNA tends to be further from the centre of the nuclear speckle, leading to the hypothesis that nuclear speckles have a proteinaceous core surrounded by an RNA shell [33]. By performing SIM with FISH and immunofluorescence (IF) staining, Fei *et al*. present a detailed structural layout of proteins and RNA molecules in the nuclear speckle [33].

At the nuclear-speckle core are scaffold proteins, such as SC35 and SON, and these are surrounded by RNAs such as the lncRNA MALAT1, and spliceosome components including the U1 and U2 small nuclear (sn)RNAs and U2B’’, a U2-associated protein (figure 2*e*, right) [33]. To provide an explanation for how the multi-layered organization of the nuclear speckle arises, Fei *et al*. used a computational model to replicate their observations *in silico*. Fei *et al*. found that SR domains are the major determinant of nuclear-speckle organization in their simulated model [33]. Specifically, favourable SR-domain interactions between SON and SC35 promote nuclear-speckle core formation, whereas interactions between SR domains and scaffold RNA molecules like MALAT1 result in the recruitment of RNAs localized to the nuclear-speckle periphery [33].

To experimentally validate their computational findings, Fei *et al*. first depleted cells of the core SR proteins and saw that nuclear speckles lost their patterned organization, confirming that SR proteins are necessary for nuclear speckle organization [33]. Next, Fei *et al*. mapped the location of several RNA transcripts encoding for nuclear-speckle-associated genes. RNA-FISH revealed that RNAs for several genes occupied a larger area than the protein core, and they found that nuclear-speckle size grows as a function of RNA occupancy, indicating that RNA accumulates in and around nuclear speckles [33]. Taken together, the findings of Fei and colleagues support a model of nuclear speckles in which RNA is concentrated and spliced at the shell, and additional processing occurs at the proteinaceous core [33,144]. How nuclear-speckle size and content influences gene expression has not been precisely characterized.

RNA is in both the nucleus and the cytoplasm. Thus, RNA-containing condensates are present in both locations. SGs are intimately associated with RNA regulation and harbour many RNA transcripts [11,76,111,112]. From a protein-focused perspective, SGs form a layered architecture with a solid core and a more liquid-like shell, but Tauber and colleagues found that, *in vitro*, addition of RNA to isolated SGs results in RNA deposition on the SG surface [111]. Based on this observation, and on prior work showing that mRNAs transiently dock onto SGs at the periphery, they wondered if RNA might be found on the surface of SGs in cells [111,112]. The researchers reasoned that if RNA is found at the surface of SGs, then an RNA helicase like eIF4A should (i) limit the amount of RNA present at the SG surface due to its unwinding activity and (ii) affect RNA–RNA interactions between condensates.

In human cells, Tauber *et al*. found that, indeed, eIF4A is localized at the periphery of SGs, and inhibiting eIF4A leads to the enrichment of several mRNAs in SGs, suggesting that the enzymatic activity of eIF4A monitors RNA at the surface of SGs [111]. Conversely, when eIF4A is overexpressed, SG formation is impaired, consistent with a model in which RNA–RNA interactions at the surface of SGs allow for proper SG maturation [111]. Thus, when RNA is considered, the model of SG architecture becomes more complex. At the protein level, scaffold proteins condense and then recruit additional proteins and RNAs [76,81,83,92]. Then, driven by the local energetic landscape, some RNAs move to the outer edge of SGs [111]. If left unchecked, these peripheral RNA molecules will interact with the RNA at the surface of adjacent SGs or other RNA–protein granules like PBs, leading to larger and larger deposits of cellular material. Therefore, RNA-remodelling enzymes, like eIF4A and other RNA helicases, represent a potential mechanism for SG regulation [111,146].

### 3.6. Nested droplets

One of the clearest examples of a biological condensate with an internal organization is the nucleolus. The nucleolus is a membraneless organelle within the nucleus and is dense with protein: over 700 protein species reside in the human nucleolus [147–150]. The nucleolus plays a critical role in the cell, as it is the site of ribosome biogenesis [147,148,151,152]. Moreover, the nucleolus is also involved in the cell cycle, stress response, and in processing RNA [148,151,153]. The three major roles of the nucleolus associated with ribosome generation are rDNA transcription, rRNA processing and ribosomal RNP assembly [147,148,153]. In elegant fashion, these three steps are spatially distinct, leading to a tripartite organization composed of a fibrillar centre (FC), a dense fibrillar component (DFC) and a granular component (GC) (figure 2*f*) [88,153,154]. The FC holds components of RNA Pol I necessary for transcription, whereas the DFC surrounding it contains proteins for pre-rRNA processing, including fibrillarin (FIB1) [88,147,151]. Collections of FCs and DFCs are, in turn, embedded within the GC, which is enriched for proteins involved in assembling the ribosome, such as NPM1 [88,154]. Thus, the interior organization of nucleoli is distinct from that of the core-shell architecture of other condensates discussed thus far. For nucleoli, the less dense exterior GC is made of a specific set of proteins that is functionally and spatially separated from the proteins found in the FC and DFC.

Interestingly, each of these three substructures within the nucleolus exhibits liquid-like behaviour. Using the large nucleoli found in *X. laevis* oocytes, Feric *et al*. [88] demonstrate that the nucleolus as a whole has liquid-like characteristics (e.g. droplet fusion), and each of the FC, DFC and GC have immiscible liquid-like properties. The precise physical nature of each of these components, however, is distinct. Using FRAP, Feric *et al*. show that at the outermost layer, the GC, has fast dynamics that are typical of liquids [88]. On the other hand, the DFC within the GC has slower recovery dynamics, indicative of a material that has more viscous character [88].

Why are these protein condensates immiscible? The more viscous behaviour of the DFC is due, in part, to the RNA-methyltransferase domain (MD) at the C-terminal end of FIB1. FIB1 has a disordered N-terminal arginine (R)/glycine (G)-rich domain which can phase separate on its own *in vitro* and is much more liquid-like than its full-length counterpart [88]. However, when this isolated RG-domain is in the cellular environment, spatial segregation is lost and the RG-domain of FIB1 is diffuse throughout the nucleolus [88]. Thus, the RNA-interacting MD of FIB1 is required for the integrity of the DFC within the nucleolus. Whether the ability to drive DFC formation is inherent to FIB1 itself or is related to its RNA-binding capability is still an open question. NPM1 also has an N-terminal oligomerization domain and a C-terminal RRM, both of which are required for *in vitro* phase separation [88]. In the cell, deletion of the RRM of NPM1 again results in aberrant spatial organization, with NPM1 found throughout the nucleolus [88]. Hence, the RBDs of both FIB1 and NPM1 are important for establishing the internal spatial architecture of the nucleolus, raising the possibility that RNA may contribute to the formation and patterning of nucleolar substructures.

### 3.7. Non-spherical condensates

Each of the examples of condensate architecture discussed thus far can be approximated as spheres. However, condensates need not be spherical. For example, the TIGER (TIS granule-ER) domain forms a mesh-like network of tubules that is woven around the ER (figure 2*g*) [89,155]. Ma & Mayr first identified the TIGER domain by studying TIS11B, an RBP found at the surface of the ER. Using FRAP in live cells, Ma & Mayr determined that TIS11B condensates are gel-like, as the fluorescence recovery was slow, and proteins associated with TIS11B granules are much less dynamic than their cytoplasmic counterparts. TIS11B preferentially binds RNA with AU-rich elements (AREs), and the presence of AREs in the 3'UTR of an mRNA is required for the association of RNA with TIS11B at the ER. Curiously, mRNAs with AREs that encode transmembrane domains are preferentially incorporated into TIGER domains, suggesting that association with the TIS11B network has functional implications for protein localization. TIGER domains also incorporate additional proteins, including HuR, which also binds to RNAs with AREs, and protein chaperones like Hsc70 and NACA, a ribosome-associated chaperone [89].

The sprawling network of the TIGER domain is striking. To gain a better understanding of how TIS11B granules form, Ma *et al*. [156] investigated the role of RNA in shaping condensates. Mutations to the RBD of TIS11B leads to the formation of sphere-like condensates, whereas replacing the RBD of TIS11B with the structurally distinct RRMs from TIGER-resident protein HuR recovered the formation of a mesh-like network. Together, these findings indicate that the distinctive organization of the TIGER domain is related to binding particular RNAs. Furthermore, RNA binding is important for constructing the network of tubules seen for the full-length protein, as fusing the TIS11B RBD (TIS^RBD^) to either the multi-valent SUMO-SIM scaffold or the PrLD of FUS (FUS^IDR^) replicated the protein–RNA network [156]. The mesh-like architecture is also not dependent on the ER, because SUMO-SIM-TIS^RBD^ forms a tubule network that is not intertwined with the ER [156].

*In vitro*, purified recombinant FUS^IDR^-TIS^RBD^ fusions form spherical condensates [156]. However, when this chimeric protein was incubated with 3'UTRs from transcripts known to associate with the TIGER domain, Ma and colleagues were able to recapitulate the gel-like meshwork seen in cells. This observation was specific to only a subset of RNAs. Notably, for any single pro-network RNA, mesh formation required an RNA concentration higher than what is typically found in the cytoplasm. However, lower concentrations of multiple meshwork-forming RNAs can be mixed to generate a TIS network, suggesting that, in cells, collaboration between RNAs is responsible for building an extensive TIGER domain.

Characterization of the RNAs that enable TIS networks revealed that neither the GC-content nor the number of AU-rich elements in a RNA was predictive of network formation [156]. Instead, the propensity of a RNA to promote TIS networks was inversely related to how strongly an RNA is expected to form a secondary structure, with unstructured RNAs showing the highest tendency towards network formation. To test this trend experimentally, Ma *et al*. mutated a normally unstructured 3'UTR to force local base pairing. Upon forming secondary structure, this RNA lost its ability to cause TIS meshwork formation. For additional validation, the researchers compared network- and non-network-forming RNAs by native gel electrophoresis and saw that only the network-forming RNAs ran as a high molecular weight smear, indicative of a heterogeneous population of multi-meric species. Similarly, the addition of dimerization domains to non-network-forming RNAs increases the molecular multi-valency, which leads to the formation of higher-order species and induces network formation [156].

From their results, Ma *et al*. make several conclusions about the formation of non-spherical TIS condensate networks. First, TIS network formation is dependent on binding to specific unstructured RNAs [156]. Second, there is a high correlation between the number of AREs and RNA disorder, independent of RNA length, suggesting that RNAs with the intrinsic disorder are enriched at TIGER domains [156]. Third, disordered mRNAs are enriched for uridine, and transcripts with high-uridine content are more likely to encode both large and disordered proteins [156]. Thus, a model is proposed in which the TIS granule network concentrates transcripts for particularly challenging membrane-localized proteins at the surface of the ER [156]. Once localized, protein chaperones and mRNAs acting as chaperones can coordinate with translating ribosomes to fold proteins and protein complexes at their intended destination. The consequences of RNA structure on both the organization and function of biomolecular condensates is thus an intriguing area for exploration.

In addition to a tubular or meshwork-like condensate, several examples of square, layered and crystalline condensates have also been described. In *C. elegans* oocytes, RNP granule formation plays a role in organismal development [84,157], and during the course of development, these granules must be remodelled by various RBPs. However, when the DEAD-box helicase CGH-1 (DDX6 in humans) is deleted, proteins that normally form spherical condensates assemble into square, sheet-like granules [157,158]. Similarly, the deletion of the *Drosophila* LSm protein, Tral, in fly oocytes also affects condensate formation, inducing more crystalline, less dynamic structures [159]. Together, these studies demonstrate the heterogeneity of condensate morphology, and reinforce the hypothesis that RBPs and RNA helicases play an essential role in the organizational maintenance of condensates [146].

Protein sequence can also have a pronounced effect on the material properties of condensates, as was recently demonstrated with the plant protein, FLOE1 [160]. FLOE1 is diffuse in seed cells that are desiccated, but when the seeds are exposed to moisture, FLOE1 forms liquid-like condensates [160]. However, when FLOE1 is mutated such that the tyrosine and phenylalanine residues in the N-terminal disordered domain are replaced with serine, instead of forming condensates that are homogeneous on the mesoscale, FLOE1 forms banded condensates with much less dynamic behaviour [160]. Thus, the presence of aromatic residues can have a marked impact on the form and function of biomolecular condensates. Research into the properties and structure of condensates in plants is a growing field, and further work may prove fruitful in designing drought-resistant crops or plants that can more efficiently capture greenhouse gases [160].

Liquid crystal condensates can also be a critical material state in biological processes. Specifically, the synaptonemal complex (SC), which joins paired meiotic chromosomes in reproductive cells, is a layered liquid crystal formed by the LLPS of several proteins [161]. The structure of the SC is sensitive to 1,6-hexanediol treatment as well as salt concentration, indicating that SC proteins coacervate due to hydrophobic and ionic interactions [161]. Thus, the SC is adapted to respond to a host of environmental factors that may hamper reproduction, providing an important check on cell cycle progression [161]. Moreover, many proteins involved in key meiotic events, including crossover between sister chromatids, are spatially constrained within the SC, suggesting that liquid crystal condensates also act to organize biological processes [161,162].

### 3.8. Non-liquid condensates

In addition to condensates with liquid-like behaviour, there are many examples of functional structures exhibiting solid, gel or glass-like properties [90,124,163–171]. And, while non-liquid condensates do not necessarily exhibit the same type of internal architecture as more liquid-like condensates, they often are formed by the specific arrangement of their component molecules [124,165]. One notable example is the Balbiani body, a membraneless organelle found in oocytes that houses membrane-bound organelles, and is involved in maintaining germline identity with potential roles in preserving organelle integrity during oocyte dormancy (figure 2*h*) [90]. In *X. laevis* oocytes, Balbiani bodies are replete with the intrinsically-disordered protein, Xvelo, which has a PrLD and a putative RBD [90]. Xvelo is uniquely able to associate with Balbiani bodies, as other RBPs with PrLDs do not localize to Balbiani bodies, or do so in a transient, non-specific manner [90]. Furthermore, unlike other RBPs, Xvelo forms a solid-like ThT-positive matrix *in vitro* and in oocytes, a phenomenon which depends on the presence and identity of its PrLD [90]. This Xvelo netting recruits both RNA and mitochondria, which likely reflects its functional role within the larger Balbiani body [90,170].

Although the transition from liquid-like to solid-like condensates can be associated with disease [9,53,55,94,101,119], some proteins can function in both liquid and solid condensates. TDP-43, for example, associates with a variety of liquid-like RNP granules [65], but it can also form solid-like myo-granules in muscles [169]. During healthy muscle regeneration, Vogler *et al*. observed that TDP-43 forms higher-order oligomers in the cytoplasm of new muscle cells before becoming exclusively nuclear upon maturation [169]. These TDP-43 inclusions are distinct from SGs and form an amyloid-like structure that displays β-sheet character [169]. Furthermore, TDP-43 myo-granules are highly enriched for sarcomere-associated RNA transcripts, which is distinct from the RNA-binding pattern observed in other cell types, such as neurons [169]. However, myo-granules can also seed pathological amyloid formation, and thus abnormalities in cellular regeneration may contribute to degenerative diseases [169,172].

## 4. Functional examples of organized condensates

Phase-separated biomolecular condensates drive diverse functions, including T-cell receptor signalling and stress signalling, actin assembly, skin barrier formation, eye lens formation and pattern development [173–179]. Thus, researchers have become interested in elucidating what role the internal organization of condensates plays in biology. Here, we describe examples of functional biomolecular condensates for which internal structure facilitates activity.

### 4.1. Ubiquitination of gene-body nucleosomes

Recently, it has been discovered that several enzymatic processes rely on the formation of biomolecular condensates with specific organizations, including the monoubiquitination of histones [47]. The polyubiquitination of proteins is typically associated with downstream degradation by the proteasome, but in the nucleus, monoubiquitination of histones contributes to effective transcription [180,181]. Monoubiquitination occurs across the genome, but how this modification is established globally was unclear. In yeast, monoubiquitination of histone H2B at lysine 123 (H2BK123) requires Uba1, Rad6 and Bre1 [182–185]. Therefore, Gallego *et al*. [47] set out to understand the mechanism by which large-scale ubiquitination of gene-body nucleosomes occurs. They identified a biomolecular condensate with a liquid core that is required to effectively ubiquitinate histone H2B.

To begin to understand how H2BK123ub is deposited on a large scale, Gallego *et al*. [47] focused on additional factors that associate with the ubiquitination machinery. An intrinsically disordered yeast protein, Lge1, had been found to copurify with an E3 ubiquitin-protein ligase Bre1 [186]. This finding led the researchers to ask if Lge1 was involved in the ubiquitination of H2B. Gallego and colleagues determined that Lge1 promotes the oligomerization of Bre1 via phase separation. However, Bre1 does not undergo LLPS on its own, so Gallego *et al*. hypothesized that the disordered domains of Lge1 allow it to act as a scaffold protein that can phase separate and recruit Bre1 as a client. *In vitro* imaging of mGFP-Bre1 added to Lge1 condensates shows that Bre1 localizes to the outside of the liquid Lge1 condensates [47]. At the core, Lge1 remains liquid-like, whereas the outer shell is decorated with Bre1 [47].

To explore the functional role of these organized condensates in ubiquitination, fluorescently tagged Rad6 (an E2 ubiquitin-conjugating enzyme) was added to pre-formed Lge1-Bre1 condensates. Gallego *et al*. observed enrichment of Rad6 at the outer shell of the Lge1 condensates in the presence of Bre1. Although these experiments were done in the absence of nucleosomes, the results suggest that the Lge1 condensate could serve as a scaffold to concentrate the Bre1-Rad6 complex at the site of nucleosomes to promote monoubiquitination. Furthermore, full-length Lge1 accelerates H2B ubiquitination of reconstituted yeast histones *in vitro*, and the IDR of Lge1 is required for both protein condensation and cell viability. These findings suggest that Lge1 LLPS is essential for promoting extensive H2B ubiquitination. Gallego *et al*. therefore propose that the phase-separated liquid-like core of Lge1 recruits Bre1 at its exterior, where it can then form a catalytic shell with Rad6, accelerating the ubiquitination of H2B [47]. In this case, the scaffold protein identifies a site of activity, and its condensation recruits and allows for oligomerization of client enzymes at the periphery of the condensate. LLPS of Lge1 and Bre1 in this manner significantly promotes ubiquitination at gene-body nucleosomes, and failure to establish this core-shell organization may have functional consequences. In fact, mutations in WAC (the human analogue of Lge1) are linked to a rare neurodevelopmental disorder in humans, DeSanto-Shinawi syndrome [187,188]. Histone monoubiquitination is a clear example of biomolecular condensates promoting cellular function by spatially clustering the necessary components for a particular action. However, whether these condensates physically bind to gene bodies, and how such location-specific binding is determined, was not revealed and are interesting questions to pursue. Several recent studies have suggested that controlling access to the genome is related to the LLPS of many different molecules [189–195], and so how biomolecular condensates and their architecture contribute to chromatin organization over time is an area of open investigation.

### 4.2. RNA interference and epigenetic inheritance

Biomolecular condensates also function in the form of associated droplets. For example, a tri-droplet condensate forms during embryogenesis in *C. elegans* to facilitate heritable RNA interference (RNAi). RNA interference is a process by which certain transcripts are silenced by dsRNA, and in some organisms, including *C. elegans*, RNAi is passed down through generations [196–201]. To investigate the means by which RNA facilitates transgenerational epigenetic inheritance (TEI), Wan *et al*. [202] describe a mechanism in which RNAi TEI is conferred by interactions between a three-component condensate assembly of proteins and silencing RNAs. First, Wan and colleagues performed a genetic screen in *C. elegans* to identify proteins required for RNAi inheritance. One component they identified was ZNFX-1, an RNA helicase present in most eukaryotic genomes [203]. Indeed, when ZNFX-1 KO animals were treated with a known RNA silencer, oma-1 dsRNA, progeny failed to inherit RNA silencing [202].

As with ZNFX-1, deletion of WAGO-4, a member of an Argonaute protein family associated with RNA silencing and inheritance, also abolished heritable RNA silencing [202]. While studying ZNFX-1 and WAGO-4, Wan *et al*. discovered that both proteins colocalize to P granules during embryogenesis and later establish separate, novel condensates, which they term Z granules [202]. The discovery of Z granules led the authors to hypothesize that these proteins work together to transmit RNAi inheritance.

Z granules exhibit dynamic properties when tested by FRAP analysis, indicating a condensed liquid phase. In adult germ cells, in addition to stand-alone Z granules, the researchers also observed Z granules sandwiched between two other phase-separated structures, P granules and Mutator foci, forming what they term PZM assemblages [202]. The order of the PZM tri-droplets may be important for RNA-based TEI, as for every PZM assembly observed, Z granules always bridged the space between P granules and Mutator foci. Furthermore, defective P granules disrupt Z granule morphology and ZNFX-1 fails to bind RNA involved in TEI [202]. In sum, these data suggest that specific spatial and temporal organization of liquid-like condensates may serve to guide RNAi inheritance pathways through the recruitment and positioning of proteins and RNAs within the cell.

Z granules also exhibit their own internal spatial organization. Wan *et al*. [204] identified the gene, Z granule surface protein-1 (*zsp-1*), as being essential for Z granule formation. The *zsp-1* gene is translated in the germline of *C. elegans* and forms puncta around the germ cell nuclei. Upon more detailed characterization of ZSP-1 localization using stimulated emission depletion microscopy, the authors found that ZSP-1 localized to the exterior of Z granules [204]. Moreover, proper ZSP-1 localization is critical for maintaining the liquid-like properties of the Z granule. Interestingly, this finding is reminiscent of the internal organization of P granules, one of the three components in PZM complex. However, ZSP-1 and the P granule surface protein, MEG-3 have very different relationships with their respective condensates [84,116,204]. Nevertheless, in future studies, it will be of interest to determine if ZSP-1 and the P granule surface protein, MEG-3, interact in a bridge or node-based network, and whether this network is involved in TEI.

### 4.3. Nucleolus phase dynamics

The coexistence of three condensates working together to facilitate function also occurs in the nucleolus, where a three-component condensate drives ribosome biogenesis. For ribosomes to be formed correctly, rRNA must be processed in a standardized manner. Because the nucleolus is critical for ribosomal assembly, nucleolar organization may thermodynamically regulate the flow of rRNA and ensure proper ribosome biogenesis [32,205]. Nascent rRNA molecules emerging from the FC and DFC are not yet bound to ribosomal proteins, so they can readily interact with the proteins housed in the outermost granular component of the nucleolus, such as NPM1 [32]. Conversely, rRNA molecules bound to ribosomal proteins (e.g. 30S and 70S rRNAs) can no longer interact with nucleolar proteins, and are thus released into the nucleoplasm.

To explore the possibility that rRNAs interact differentially with the nucleolus as a function of their maturity, the partitioning of 16S, 30S or 70S rRNA with NPM1 condensates was compared [32]. The least mature 16S rRNA strongly partitioned into NPM1 condensates, whereas the protein-bound 30S and 70S rRNAs were largely excluded [32]. These data support a model whereby nascent rRNAs are transcribed and initially processed in the FC and DFC, then bind first to NPM1 in the GC. Next, as rRNA : NPM1 interactions are replaced with rRNA : ribosomal protein interactions, it is no longer thermodynamically favourable for the maturing ribosomal subunits to remain in the nucleolus, and they are released into the nucleoplasm [32]. In such a schema, the internal organization of the nucleolus serves as a thermodynamic assembly line to control ribosome biogenesis. This hypothesis for ribosome biogenesis serves as an attractive framework for conceptualizing future studies to understand the finer mechanistic details of nucleolar activity and other condensate-based processes.

## 5. Concluding remarks

Early advances in the research on LLPS in biology relied on simplified *in vitro* systems containing a limited number of molecules under very specific environmental conditions. However, it has become apparent that the nature of biomolecular condensates in living organisms is highly nuanced and dynamic, allowing these bodies to respond to a changing cellular milieu. One way by which such rapid activity could be facilitated is if condensates themselves have some degree of organization. Indeed, there are now many examples where condensed mixtures of proteins and nucleic acids adopt an ordered internal architecture. Furthermore, such organization can be understood on both a fundamental physical and chemical basis, as well as on a larger functional scale. In this review, we have focused on how and why complex biomolecular condensates adopt specific internal organization and catalogue the condensate architectures described thus far in the literature. As research progresses in the field of biomolecular condensates, we expect that both the number and variety of condensate architectures will continue to grow, and we hope that our review can serve as a guide for understanding internal condensate organization.
